# Supplementary material for: Role and mechanism of NCAPD3 in promoting malignant behaviors in gastric cancer
Source: Front Pharmacol. 2024 Apr 22;15:1341039. doi: 10.3389/fphar.2024.1341039 (PMC11070777; doi:10.3389/fphar.2024.1341039)
Supplement: Supplementary file 11 [file DataSheet2.ZIP › GSEA/GO_CC/my_analysis.Gsea.1599461408869/GO_MITOCHONDRIAL_PROTEIN_COMPLEX.html]

Details for gene set GO\_MITOCHONDRIAL\_PROTEIN\_COMPLEX[GSEA]

|  || Dataset | filtered\_dataset.sample\_info.cls#WT\_versus\_NCAPD3\_MUT |
| Phenotype | sample\_info.cls#WT\_versus\_NCAPD3\_MUT |
| Upregulated in class | WT |
| GeneSet | GO\_MITOCHONDRIAL\_PROTEIN\_COMPLEX |
| Enrichment Score (ES) | 0.3554643 |
| Normalized Enrichment Score (NES) | 1.4267142 |
| Nominal p-value | 0.10412926 |
| FDR q-value | 0.6853657 |
| FWER p-Value | 0.997 |
Table: GSEA Results Summary

  

Fig 1: Enrichment plot: GO\_MITOCHONDRIAL\_PROTEIN\_COMPLEX      
 Profile of the Running ES Score & Positions of GeneSet Members on the Rank Ordered List

  

| SYMBOL | TITLE | RANK IN GENE LIST | RANK METRIC SCORE | RUNNING ES | CORE ENRICHMENT || 1 | 196294 | IMMP1L | 216 | 0.662 | -0.0640 | Yes |
| 2 | 91574 | C12orf65 | 238 | 0.646 | 0.0096 | Yes |
| 3 | 87178 | PNPT1 | 263 | 0.622 | 0.0779 | Yes |
| 4 | 54931 | TRMT10C | 446 | 0.507 | 0.0170 | Yes |
| 5 | 617 | BCS1L | 513 | 0.467 | 0.0338 | Yes |
| 6 | 29090 | TIMM21 | 537 | 0.453 | 0.0796 | Yes |
| 7 | 78988 | MRPL57 | 557 | 0.441 | 0.1266 | Yes |
| 8 | 55735 | DNAJC11 | 625 | 0.409 | 0.1347 | Yes |
| 9 | 10452 | TOMM40 | 696 | 0.380 | 0.1367 | Yes |
| 10 | 55750 | AGK | 697 | 0.380 | 0.1889 | Yes |
| 11 | 6182 | MRPL12 | 729 | 0.359 | 0.2160 | Yes |
| 12 | 64087 | MCCC2 | 747 | 0.352 | 0.2521 | Yes |
| 13 | 79590 | MRPL24 | 800 | 0.320 | 0.2588 | Yes |
| 14 | 51373 | MRPS17 | 817 | 0.306 | 0.2893 | Yes |
| 15 | 516 | ATP5G1 | 826 | 0.301 | 0.3249 | Yes |
| 16 | 6183 | MRPS12 | 838 | 0.280 | 0.3555 | Yes |
| 17 | 5163 | PDK1 | 982 | -0.394 | 0.3070 | No |
Table: GSEA details [plain text format]

  

Fig 2: GO\_MITOCHONDRIAL\_PROTEIN\_COMPLEX      
 Blue-Pink O' Gram in the Space of the Analyzed GeneSet

  

Fig 3: GO\_MITOCHONDRIAL\_PROTEIN\_COMPLEX: Random ES distribution      
 Gene set null distribution of ES for **GO\_MITOCHONDRIAL\_PROTEIN\_COMPLEX**
